# Disitamab Vedotin (RC48) for HER2-positive advanced breast cancer: a case report and literature review

**DOI:** 10.3389/fonc.2023.1286392

**Published:** 2023-11-24

**Authors:** Yang Li, Jingjiao Zhang, Zhengang Cai, Xue Gao, Lina Zhang, Zhi Lu, Xiaojie Wang, Peiyao Yu, Jia Li, Fengqi Fang

**Affiliations:** ^1^ Department of Oncology, First Affiliated Hospital of Dalian Medical University, Dalian Medical University, Dalian, China; ^2^ Department of Breast Surgery, First Affiliated Hospital of Dalian Medical University, Dalian Medical University, Dalian, China; ^3^ Department of Pathology, First Affiliated Hospital of Dalian Medical University, Dalian Medical University, Dalian, China; ^4^ Imaging and Nuclear Medicine Department, First Affiliated Hospital of Dalian Medical University, Dalian Medical University, Dalian, China; ^5^ Nuclear Medicine Department, First Affiliated Hospital of Dalian Medical University, Dalian Medical University, Dalian, China; ^6^ Department of Radiotherapy, First Affiliated Hospital of Dalian Medical University, Dalian Medical University, Dalian, China

**Keywords:** Disitamab Vedotin (RC48), advanced breast cancer, human epidermal growth factor receptor 2, targeted therapy, tumor resistance, case report

## Abstract

**Background/aim:**

Human epidermal growth factor receptor 2 (HER2)-positive breast cancer is associated with a higher risk of metastasis and poorer overall survival (OS) due to HER2 gene overexpression/amplification. Although anti-HER2 targeted therapy has shown survival benefits in HER2-positive advanced breast cancer (ABC) patients, long-term treatment often leads to drug resistance, complicating further treatment options. RC48, an antibody-drug conjugate (ADC), combines the benefits of antibody targeting with the cytotoxic effects of a small molecule drug.

**Case report:**

We present a case involving a female patient with HER2-positive ABC who developed drug resistance and disease progression following multi-line anti-HER2 targeted therapy. In this instance, RC48 exhibited anti-tumor activity in an ABC patient resistant to HER2-targeted therapy. After eight treatment cycles with 120 mg of RC48, the tumor size decreased and stabilized.

**Conclusion:**

This case report underscores the potential clinical value of RC48 as a promising treatment alternative for patients resistant to HER2 targeted therapies.

## 1 Introduction

Breast cancer is the most common malignant tumor worldwide, with the highest incidence and the second highest mortality in women (1). Human epidermal growth factor receptor 2 (HER2)-positive breast cancer represents approximately 15-20% of all breast cancer cases. This tumor subtype, HER2-positive breast cancer, is more invasive, prone to metastasis, and associated with poorer prognosis (2). Anti-HER2 therapy can greatly improve the survival rate of patients; however, after multi-line treatment, the majority of advanced breast cancer (ABC) patients eventually develop drug resistance, leading to disease progression, and the limited subsequent treatment options (3). Currently, the mechanisms underlying resistance to anti-HER2 targeted therapy are under study. Research has indicated that resistance may be associated with spatial effects (structural mutations of the HER2 protein), overexpression of tyrosine kinase receptors [such as the insulin-like growth factor receptor (IGFR)], and mutations in the HER2 downstream signaling pathway (4). Overcoming resistance to anti-HER2 targeted therapy is crucial, and identifying new effective treatment strategies can provide valuable insights for clinical practice.

Currently, the primary treatment for HER2-positive ABC involves anti-HER2 targeted therapy drugs, often utilizing a combination of trastuzumab and pertuzumab as first-line treatment (3). The combination of trastuzumab and pertuzumab synergistically enhances the inhibition of the HER2 pathway (5).

The study of von Minckwitz et al. (6) showed that the HER2 pathway could be further inhibited when the first-line treatment disease progresses, and subsequent targeted therapy still has application value. Trastuzumab emtansine (T-DM1) is a novel antibody-drug conjugate (ADC). Verma et al. (7) assessed the utility of T-DM1 in patients who experienced disease progression following trastuzumab treatment. The T-DM1 treatment group had an objective response rate (ORR) of 43.6%, a median progression-free survival (PFS) of 9.6 months, and overall survival (OS) of 30.9 months, with *P* < 0.0017. These results indicate that T-DM1 could serve as a preferred drug for targeted therapy after drug resistance develops. DS-8201 (T-DXd) is another novel ADC drug. In a phase III clinical trial (NCT03529110), T-DXd treatment demonstrated significant OS and PFS benefits (28.8 months vs. 6.8 months) compared to T-DM1, and T-DXd substantially reduced the risk of death by 36% (HR, 0.64) (8).

Disitamab Vedotin (RC48) is an ADC with a higher affinity for HER2 than the targeted drug trastuzumab (KD, 5.0E-10M vs KD, 1.9E-09M) (9). By utilizing antibodies, RC48 can block the downstream signaling pathway activated by HER2, work in conjunction with cytotoxic drugs to kill tumor cells and interfere with the transcription, division, proliferation, and growth of cancer cells to exert anti-tumor effects. In a clinical trial (NCT02881190), RC48 demonstrated stronger antitumor activity in HER2-positive breast cancer, gastric cancer, and trastuzumab and lapatinib-resistant xenograft tumor models than the FDA-approved T-DM1 (10, 11).

However, to the best of our knowledge, there have been limited studies reporting the efficacy of RC48 monotherapy in ABC patients resistant to HER2-targeted therapy. Therefore, we present a case of a female patient with HER2-positive ABC who developed resistance to multiple lines of anti-HER2 therapy. This patient experienced significant benefits and ultimately became eligible for surgery following treatment with RC48. This case report offers new insights into addressing resistance to HER2 targeted therapy in clinical practice.

## 2 Case report

A 54-year-old female patient was admitted to an external hospital on December 15, 2019 due to the discovery of a right breast tumor for 2 weeks. A physical examination revealed a tumor in the upper quadrant of the right breast. Ultrasound-guided right breast tumor biopsy pathology indicated non-specific invasive breast cancer in the right breast; immunohistochemical staining results showed that estrogen receptor (ER) (70% weak-moderate intensity +), progesterone receptor (PR) (40% weak-moderate intensity +), HER2 (3 +), proliferation index (Ki67) (80%); right axillary lymph node biopsy cytology showed cancer metastasis. The patient was diagnosed with right breast invasive breast cancer (cT4N1M0 stage III). The patient began anti-tumor treatment in July 2020 after half a year, and had been treated with multiple lines. The treatment is shown in **Table 1**.

**Table 1:** Treatment of HER2-positive ABC patients.

Time range	Treatment lines	Treatment regimen	Best response	PFS (months)
**Jul 2020 — Aug 2020**	Neoadjuvant	Albumin Paclitaxel + Fluorouracil X2	PD	1.5
**Sept 2020— Nov 2020**	Neoadjuvant	Albumin Paclitaxel + Carboplatin + Trastuzumab + Patuzumab X4	PR	12
**Dec-2020 — Sept 2021**	N/A	The patient discontinued treatment due to economic factors	N/A	N/A
**Sept 2021 — Dec 2021**	First-line	Albumin paclitaxel + capecitabine + trastuzumab + pertuzumab X2; Docetaxel + trastuzumab + pertuzumab X2	PD	3
**Dec 2021 — Mar 2022**	Second-line	Capecitabine + pyrotinib + trastuzumab + pertuzumab X4	PD	3
**Mar 2022 — Jun 2022**	Third-line	Inetetamab + Vinorelbine + cisplatin X2	PD	2
**Jul 2022 — Oct 2022**	Fourth-line	RC48 (2.0mg/kg) 120mg X8 (July 6th—Oct 21st)	PR	4
**Nov 2022**	Surgery	Modified radical mastectomy of right breast	NA	NA
**Nov 2022 — Today**	Maintenance	Trastuzumab + pyrotinib + abemaciclib + fulvestrant	SD	8

PD, progressive disease; PR, partial remission; SD, stable disease; NA, not applicable.

On July 14, 2020, the patient began neoadjuvant chemotherapy, receiving two cycles of albumin-bound paclitaxel combined with fluorouracil (specific dose: albumin-bound paclitaxel 125mg/m2 d1 d8 ivgtt, 5FU 500mg/m2 d1 ivgtt, with 21 days per cycle), during which the tumor continued to grow. On August 27, 2020, the patient underwent a PET-CT examination, which revealed right breast cancer with right axillary lymph node metastasis and a response of progressive disease (PD).

**Figure 1:**
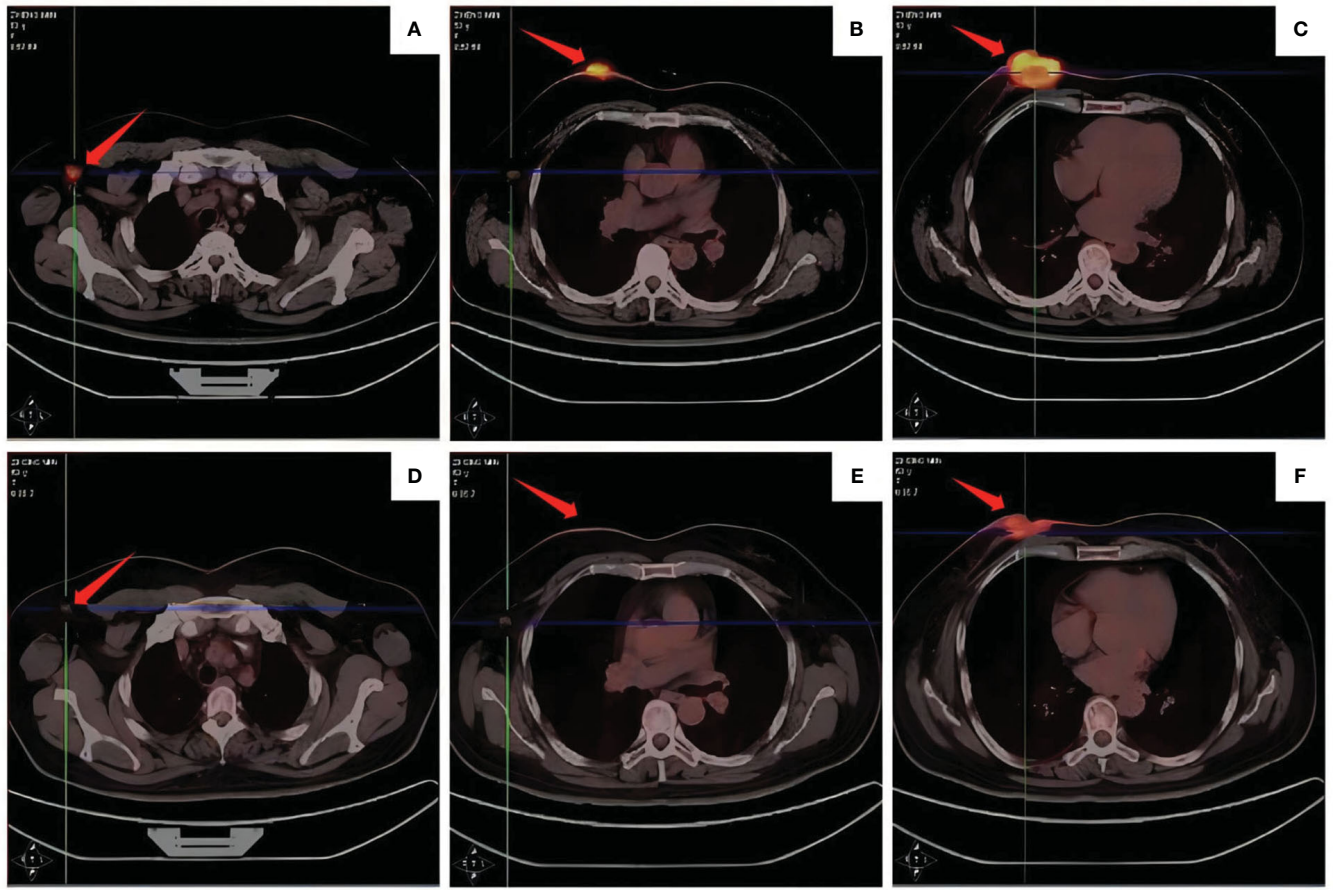
PET-CT results of patients at different time. On August 27, 2020, **(A, B)** multiple lymph nodes in the right axilla were enlarged, with a large diameter of about 1.5 cm, SUVmax: 9.1; **(C)** 3.7 cm x 3.8 cm tumor was seen in the right breast nipple, SUVmax: 22.4; **(D-F)** after four cycles of treatment (November 20, 2020), the right breast tumor and axillary lymph nodes were smaller than before.

On September 1, 2020, the neoadjuvant treatment regimen was changed to albumin-bound paclitaxel, carboplatin, and trastuzumab for two cycles (actual dose: albumin-bound paclitaxel 200mg d1, d8 ivgtt, carboplatin 700mg d1 ivgtt, trastuzumab 580mg for the first dose, followed by 430mg ivgtt d1 q21d). Since the tumor did not shrink after two chemotherapy cycles, pertuzumab (initially 840mg, followed by 420mg ivgtt d1) was added for two cycles. After completing four treatment cycles, the PET-CT findings from November 20, 2020, were juxtaposed with the data from August 27, 2020, for comparative analysis. The right breast tumor had reduced in size, and the right axillary lymph node had also decreased in size (**Figure 1**), achieving the best response of partial response (PR). For economic reasons, the patient discontinued treatment on their own accord.

In September 2021, the right breast tumor locally enlarged and ulcerated. A chest CT scan revealed multiple enlarged lymph nodes in the bilateral axilla, which were larger and more numerous than before. On September 27, 2021, the patient began first-line treatment with two cycles of albumin-bound paclitaxel, capecitabine, trastuzumab, and pertuzumab (actual dose: albumin-bound paclitaxel 200mg d1, d8 ivgtt, capecitabine 1000mg/m2 d1-d14 bid po, trastuzumab 430mg ivgtt d1 q21d, pertuzumab 420mg ivgtt d1). Due to economic factors, the chemotherapy drug was switched to docetaxel, and the actual medication regimen was docetaxel, trastuzumab, and pertuzumab for two cycles. After four cycles of treatment, the tumor was visibly larger, and the bleeding became severe. A chest CT scan showed that the bilateral axillary lymph nodes had increased in size compared to before, with a response of PD.

Due to disease progression following first-line treatment, the patient underwent second-line treatment on December 27, 2021, with four cycles of capecitabine, pyrotinib, trastuzumab, and pertuzumab (actual dose: capecitabine 1000mg/m2 d1-d14 bid po, pyrotinib 400mg qd po, trastuzumab 430mg ivgtt d1 q21d, pertuzumab 420mg ivgtt d1). During treatment, the tumor ruptured, and bleeding was stopped using an external application of Yunnan Baiyao powder. On March 17, 2022, a chest CT scan was reviewed, showing that the bilateral axillary lymph nodes had increased in size compared to before, with a response of PD. Considering the possibility of drug resistance, the patient was advised to undergo genetic testing. The results revealed a PIK3CA exon 10 c.1624G>A p. E542K missense mutation, but related targeted drugs were not yet available to us.

On March 18, 2022, the patient underwent third-line chemotherapy with two cycles of Inetetamab, vinorelbine, and cisplatin (specific drugs: Inetetamab 550mg ivgtt d1 q21d, vinorelbine 40mg ivgtt d1, d5 q21d, cisplatin 60mg ivgtt d1 q21d). On June 9, 2022, PET-CT results showed that the original right breast tumor had significantly increased in size compared to before, and the original right axillary lymph nodes were larger and more numerous than before. New lymph nodes with increased FDG metabolism appeared in the bilateral chest wall, right internal mammary region, and left axilla, indicating disease progression once again, with a response of PD.

**Figure 2:**
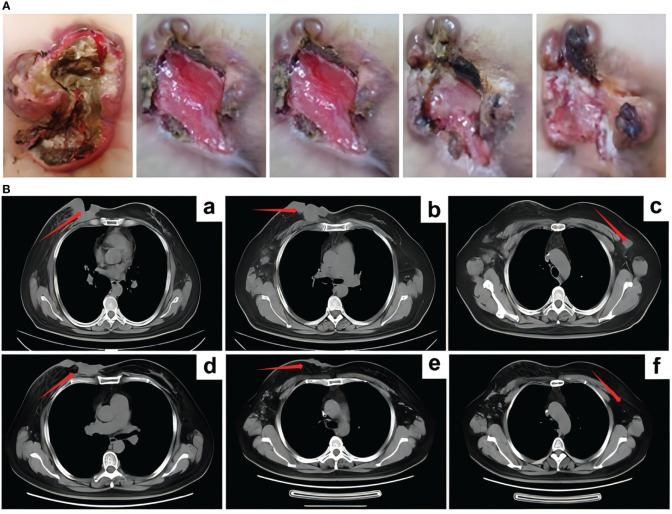
The curative effect of RC48 treatment. **(A)** After RC48 treatment, the tumor rupture was smaller than before. **(B)** CT efficacy evaluation after 6 cycles of RC48 treatment. (a-c) Patient status before RC48 treatment; (d-f) after RC48 treatment, the breast tumor and bilateral axillary lymph nodes were significantly reduced.

On June 16, 2022, a right axillary lymph node biopsy revealed poorly differentiated carcinoma. Immunohistochemistry results were as follows: ER (3 + 80%), PR (–), HER2 (3+), and Ki-67 (+ 2%). The immunohistochemical results confirmed that the patient still had HER2 overexpression. Considering the chemotherapy and targeted resistance after previous multi-line chemotherapy, the patient was given RC48 as a fourth-line treatment, which is administered in a cycle every 14 days with the treatment being given on the first day of each cycle. On July 6, 2022, eight cycles of RC48 treatment were initiated, with the planned medication regimen as follows: RC48 (2.0 mg/kg) 120mg ivgtt d1 q14d. Before treatment, the patient experienced significant pain and required analgesic drugs for pain control. After RC48 treatment, the pain considerably improved, and the dosage of analgesics was significantly reduced. During the treatment period, the ulcerated tumor gradually healed (**Figure 2A**), the patient tolerated the treatment well, and no obvious adverse reactions occurred. After six cycles, a chest CT scan showed that the breast tumor and bilateral axillary lymph nodes had significantly reduced in size, with the best response of PR (**Figure 2B**).

**Figure 3:**
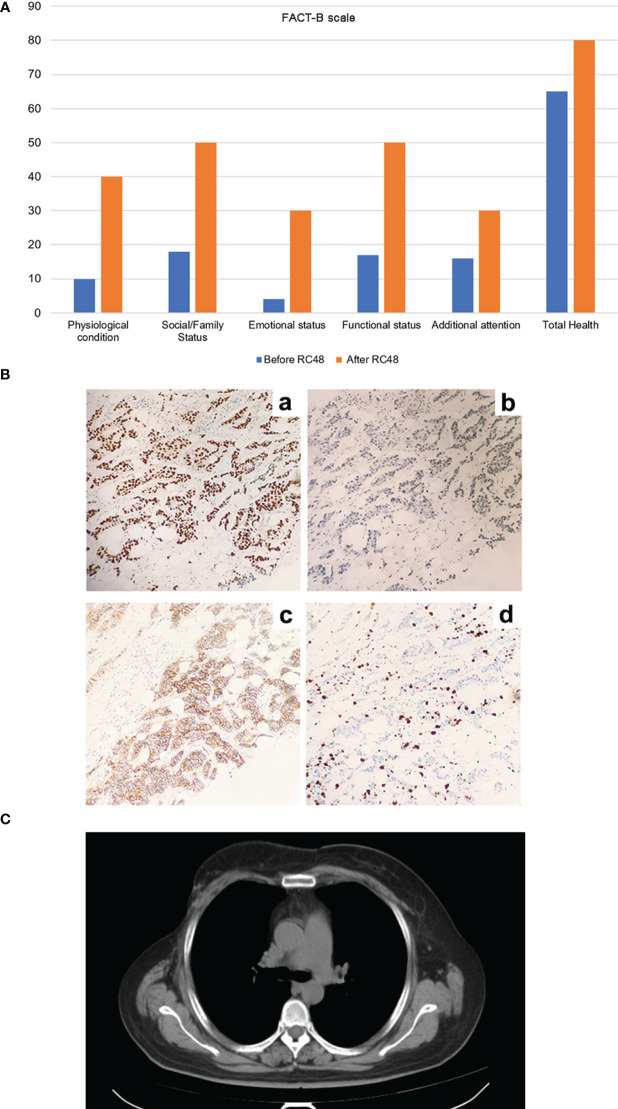
The improvement of patient’s life quality and postoperative immunohistochemical results of right breast cancer after RC48 treatment, as well as the CT results after half a year (July 12, 2023). **(A)** The five functional areas and overall health level of patients were significantly improved, and the quality of life was significantly improved. **(B)** The postoperative immunohistochemical results of right breast cancer. (a) ER (2 + 60%), (b) PR (5%), (c) HER2 (2 +), (d) Ki67 proliferation index (60%). **(C)** The patient ‘s CT results showed no enlarged lymph nodes, and the condition was stable.

In order to explore the improvement in quality of life brought about by RC48, we used the FACT-B scale to evaluate the patient-reported outcomes (PROs). After using RC48, the patient experienced significant improvements in the five functional areas and overall health compared to their previous quality of life (**Figure 3A**). In the process of multi-line anti-HER-2 treatment, the patient often has adverse reactions such as bone marrow suppression and limb end numbness, among which nausea and vomiting most affect the quality of life. During the treatment with RC48, the patient feels good about herself, no obvious systemic adverse reactions occurred, and the overall quality of life was significantly improved.

After six cycles of RC48 treatment, the tumor shrank and stabilized, with the 7th and 8th cycles continuing to show stability. In order to further improve the quality of life, a multidisciplinary team of breast cancer specialists (MDT) recommended a modified radical mastectomy of the right breast cancer, which was performed on November 21, 2022. Postoperative pathology revealed non-specific invasive carcinoma in the right breast and metastatic carcinoma in the right axillary and subclavian lymph nodes. Immunohistochemical results for the right breast lesions showed: ER (2 + 60%), PR (+ 5%), HER2 (2+), and Ki67 (60%) (**Figure 3B**); HER2 FISH was positive.

Considering that the patient’s contralateral axillary enlarged lymph node was a metastatic lymph node, it was recommended that the patient continue maintenance treatment after surgery. The patient’s postoperative pathology was positive for hormone receptor (HR+) and HER2 overexpression. The recommended treatment was trastuzumab + pyrotinib targeted therapy, combined with abemaciclib + fulvestrant targeted dual pathway endocrine therapy (specific medications: trastuzumab 430mg ivgtt d1 q21d, pyrotinib 400mg qd po, abemaciclib 50mg bid po, fulvestrant 500mg intramuscular injection). The patient’s condition was stable half a year after operation. The CT results showed that the right breast was changed after operation (**Figure 3C**).

## 3 Discussion

HER2 is a transmembrane protein with tyrosine kinase activity. It mediates signal transduction activation and downstream signaling pathways through heterodimer and tyrosine kinase autophosphorylation (12). HER2-positive tumors are prone to metastasis, and most have a poor prognosis. Anti-HER2 targeted therapy can greatly improve the survival rate of breast cancer (13). Trastuzumab combined with chemotherapy in the treatment of HER2-positive ABC patients can significantly prolong the disease-free survival (DFS) and OS. However, most patients may develop drug resistance after treatment. More than 70% of HER2-positive breast cancer patients have tumor recurrence, lymph node metastasis, etc (4).

The issue of drug resistance is crucial for patients’ prognosis. Currently, the mechanisms of targeted therapy resistance mainly involve changes in the HER2 receptor molecular structure, alterations in the PI3K/AKT/mTOR signaling pathway, and influences of the HER family on downstream signaling pathways. Other mechanisms include incomplete blocking of the HER2 signaling pathway, involvement of immune mechanisms, and changes in oncogenes/tumor suppressor genes (14–19). The patient’s genetic test suggested a missense mutation in PIK3CA, which may be the cause of drug resistance. However, due to the unavailability of related signaling pathway inhibitors, targeted treatment was not possible.

In addition, other factors affecting drug resistance and efficacy, which are clinically important, should not be overlooked. Our case report highlights a treatment gap in one patient, which may have also contributed to the observed drug resistance. Due to financial difficulties and adherence issues, our patient discontinued treatment, which may lead to a decrease in drug efficacy and the development of resistance, especially to targeted therapies such as anti-tumor necrosis factor-2 (anti-HER-2). Suspension of treatment after initial efficacy of neoadjuvant therapy may lead to the development of drug resistance in tumor cells, which may affect the efficacy of subsequent anti-HER-2 therapy. In addition, the coronavirus pandemic poses a healthcare challenge. Our patient’s treatment interruption coincided with the coronavirus epidemic. Although the treatment interruption was not directly caused by the epidemic, the economic impact may have exacerbated the patient’s financial stress, which may have influenced her decision to continue treatment.

RC48 is an antibody-coupled drug with both antibody targeting and small molecule drug killing properties. Relevant clinical studies have confirmed its efficacy in the treatment of ABC. Two phase-I clinical studies, NCT02881138 and NCT03052634 (20, 21), evaluated the efficacy of RC48 in patients with HER2-positive (IHC 3+ or IHC 2+ and FISH amplification) locally advanced or metastatic breast cancer. We performed a combined analysis of the two studies (20, 21) to assess the efficacy. Among the 70 patients treated with RC48, the ORR was 31.4% (22/70), the clinical benefit rate (CBR) was 38.6%, and the median PFS was 5.8 months. Among the 64 patients receiving ≥ 1.5 mg/kg dose, the ORR was 34.4% (22/64), and the median PFS was 6.2 months. The ORR of patients receiving 1.5 mg/kg, 2.0 mg/kg, and 2.5 mg/kg doses was 22.2%, 42.9%, and 36.0%, respectively, and the median PFS was 6.2 months, 6.0 months, and 6.3 months, respectively. It can be observed that RC48 demonstrates a favorable effect in HER2 positive metastatic breast cancer, and 2.0 mg/kg Q2W is the optimal choice. The author’s intention here is to convey an integrated analysis of the two aforementioned studies.

In another phase I clinical study (NCT02881190) (11), 57 patients with solid tumors were enrolled and treated with RC48 at a dose of 2.0 mg/kg Q2W. The ORR and disease control rate (DCR) were 21.0% (12/57) and 49.1% (28/57), respectively. Notably, the efficacy of RC48 in HER2+/FISH- patients was similar to that of IHC2+/FISH+ and IHC3+ patients, with ORRs of 35.7% (5/14), 20.0% (2/10), and 13.6% (3/22), respectively. RC48 also demonstrated promising efficacy in patients who had not previously received HER2-targeted therapy, with an ORR of 15.0% (3/20) and a DCR of 45.0% (9/20). These results suggest that RC48 may have a beneficial effect on HER2-positive or low-expressing solid tumors, further highlighting its potential as a therapeutic option for these patients.

The study by Parise C et al. (22) demonstrated that approximately 50% of HER2-positive breast cancer patients also express hormone receptors (HR), that is, HER2+/HR+ or HER2+/ER+/PR+, accounting for 10%-15% of all breast cancers. HER2 and ER-mediated signaling pathways in breast cancer patients intersect at multiple nodes. ER can affect apoptosis by activating downstream pathways such as HER2, thereby influencing the efficacy of anti-HER2 targeted therapy (23). The Monarch HER study included ABC patients who received at least two anti-HER2 regimens and those who did not receive CDK4/6 inhibitors and fulvestrant. The results showed that, compared to standard chemotherapy plus trastuzumab, abemaciclib plus trastuzumab plus fulvestrant significantly improved PFS in HER2+/ER+ ABC patients resistant to anti-HER2 therapy (8.3 months vs. 5.7 months, P=0.051) (24). According to the final OS results presented at the European Society for Medical Oncology (ESMO) in 2022 (25), abemaciclib plus trastuzumab combined with or without fulvestrant significantly improved the OS of HR+/HER2+ ABC patients compared to standard chemotherapy plus trastuzumab (31.1 months vs. 20.7 months). These findings provide guidance for our postoperative maintenance therapy.

For HER2-positive breast cancer patients, chemotherapy combined with targeted therapy is predominantly used in first-line treatment, significantly prolonging survival and improving the quality of life (26). In our case, the patient was initially diagnosed with invasive breast cancer and axillary lymph node metastasis. Immunohistochemical results showed HER2 (3+), indicating that dual-target combined chemotherapy could provide the greatest benefits (27). The patient experienced disease progression after neoadjuvant therapy and third-line anti-HER2 combined chemotherapy, and the local tumor ulceration severely impacted her quality of life. Faced with multiple drug resistance, we opted for 8 cycles of RC48 treatment. The tumor shrank significantly, the ulceration improved considerably, the patient became eligible for surgical treatment, and her quality of life improved substantially. For postoperative treatment, we employed trastuzumab + pyrotinib targeted therapy in conjunction with abemaciclib + fulvestrant maintenance therapy. This approach resulted in acceptable tumor control and a stable condition for the patient. However, if the patient experiences a relapse, determining the subsequent treatment plan will be a significant challenge.

We report the successful treatment of a HER2-positive ABC patient using RC48 monotherapy. After the failure of multiple lines of anti-HER2 treatment, the use of ADC drugs was proved effective, providing the opportunity for surgical treatment and improving the patient’s quality of life. Our case offers new therapeutic options for other ABC patients resistant to HER2 treatment, holding clinical reference value and presenting new treatment ideas for patients with refractory HER2-targeted therapy resistance.

## Data availability statement

The original contributions presented in the study are included in the article/supplementary material. Further inquiries can be directed to the corresponding authors.

## Ethics statement

The studies involving humans were approved by Ethics Committee of First Affiliated Hospital of Dalian Medical University. The studies were conducted in accordance with the local legislation and institutional requirements. The participants provided their written informed consent to participate in this study. Written informed consent was obtained from the individual(s) for the publication of any potentially identifiable images or data included in this article.

## Author contributions

YL: Conceptualization, Data curation, Methodology, Writing – original draft. JZ: Conceptualization, Software, Writing – original draft. ZC: Validation, Writing – review & editing. XG: Validation, Writing – review & editing. LZ: Validation, Writing – review & editing. ZL: Formal Analysis, Writing – review & editing. XW: Formal Analysis, Writing – review & editing. PY: Investigation, Writing – review & editing. JL: Conceptualization, Writing – review & editing. FF: Conceptualization, Resources, Supervision, Visualization, Writing – review & editing.
